# WEALTH PROFILES AND VARIATIONS BY GENDER AND RACE: A GROWTH-MIXTURE MULTIGROUP COMPARISON

**DOI:** 10.1093/geroni/igz038.2687

**Published:** 2019-11-08

**Authors:** Yu-Chih Chen, Nancy Morrow-Howell

**Affiliations:** Washington University in St. Louis, St. Louis, Missouri, United States

## Abstract

Wealth is fundamentally affected by various life course characteristics. However, little is known about the role of life course factors in shaping wealth trajectories in later life. This study explored how the longitudinal profiles of wealth varied by gender and race (white and non-white populations). Data came from the 2004-2014 Health and Retirement Study with 16,189 older adults aged 51 and older. With corrections for clustered effect within household, this study used growth mixture modeling (GMM) to identify the longitudinal patterns of wealth, and how these profiles varied by these two important life course attributes. The model began with a separate GMM model for race and gender to investigate the optimal latent class model. These results were combined using multi-group approach to incrementally examine the gender and race invariance using configural (same form), structural (same trajectory mean), dispersion (same trajectory variance), and distributional (same latent class size) test. Results identified four distinct wealth profiles—Stable high, Low and increasing, Stable low, and High but decline—for each race and gender category. The multigroup GMM analyses revealed that the wealth profiles varied by gender and race, but the degrees of variation differed a great deal, with results supporting a dispersion model for gender but a configural model for race. Results indicate that race has a stronger effect in shaping wealth development compared to gender. The findings suggest that understanding wealth disparities in later life could be facilitated by examining how wealth varies by gender and race.

